# What matters most: protocol for a randomized controlled trial of breast cancer surgery encounter decision aids across socioeconomic strata

**DOI:** 10.1186/s12889-018-5109-2

**Published:** 2018-02-13

**Authors:** Marie-Anne Durand, Renata West Yen, A. James O’Malley, Mary C. Politi, Shubhada Dhage, Kari Rosenkranz, Katie Weichman, Julie Margenthaler, Anna N. A. Tosteson, Eloise Crayton, Sherrill Jackson, Ann Bradley, Robert J. Volk, Karen Sepucha, Elissa Ozanne, Sanja Percac-Lima, Julia Song, Jocelyn Acosta, Nageen Mir, Glyn Elwyn

**Affiliations:** 1 The Dartmouth Institute for Health Policy & Clinical Practice, Geisel School of Medicine, Dartmouth College, Lebanon, NH USA; 2 Department of Surgery, Division of Public Health Sciences, Washington University School of Medicine, St. Louis, MO USA; 3 Laura and Isaac Perlmutter Cancer Center, New York University School of Medicine, New York, NY USA; 4 Dartmouth-Hitchcock Medical Center, Lebanon, NH USA; 5 Montefiore Medical Center, Bronx, NY USA; 6 Department of Surgery, Washington University School of Medicine, St. Louis, MO USA; 7 The Dartmouth Institute for Health Policy & Clinical Practice, Dartmouth College, and Norris Cotton Cancer Center, Lebanon, NH USA; 8 The University of Texas MD Anderson Cancer Center, Houston, TX USA; 9 Division of General Internal Medicine, Massachusetts General Hospital, Boston, MA USA; 10 University of Utah, Salt Lake City, UT USA; 11 Massachusetts General Hospital’s Chelsea HealthCare Center, Chelsea, MA USA

**Keywords:** Breast cancer surgery, Low socioeconomic status, Breast cancer disparities, Decision support techniques, Encounter decision aids, Picture superiority

## Abstract

**Background:**

Breast cancer is the most commonly diagnosed malignancy in women. Mastectomy and breast-conserving surgery (BCS) have equivalent survival for early stage breast cancer. However, each surgery has different benefits and harms that women may value differently. Women of lower socioeconomic status (SES) diagnosed with early stage breast cancer are more likely to experience poorer doctor-patient communication, lower satisfaction with surgery and decision-making, and higher decision regret compared to women of higher SES. They often play a more passive role in decision-making and are less likely to undergo BCS. Our aim is to understand how best to support women of lower SES in making decisions about early stage breast cancer treatments and to reduce disparities in decision quality across socioeconomic strata.

**Methods:**

We will conduct a three-arm, multi-site randomized controlled superiority trial with stratification by SES and clinician-level randomization. At four large cancer centers in the United States, 1100 patients (half higher SES and half lower SES) will be randomized to: (1) Option Grid, (2) Picture Option Grid, or (3) usual care. Interviews, field-notes, and observations will be used to explore strategies that promote the interventions’ sustained use and dissemination. Community-Based Participatory Research will be used throughout. We will include women aged at least 18 years of age with a confirmed diagnosis of early stage breast cancer (I to IIIA) from both higher and lower SES, provided they speak English, Spanish, or Mandarin Chinese. Our primary outcome measure is the 16-item validated Decision Quality Instrument. We will use a regression framework, mediation analyses, and multiple informants analysis. Heterogeneity of treatment effects analyses for SES, age, ethnicity, race, literacy, language, and study site will be performed.

**Discussion:**

Currently, women of lower SES are more likely to make treatment decisions based on incomplete or uninformed preferences, potentially leading to poorer decision quality, quality of life, and decision regret. This study hopes to identify solutions that effectively improve patient-centered care across socioeconomic strata and reduce disparities in decision and care quality.

**Trial registration:**

NCT03136367 at ClinicalTrials.gov

**Protocol version:** Manuscript based on study protocol version 2.2, 7 November 2017.

**Electronic supplementary material:**

The online version of this article (10.1186/s12889-018-5109-2) contains supplementary material, which is available to authorized users.

## Background

Breast cancer is the second leading cause of death in women [1, 2]. Despite significant improvements in overall breast cancer survival, disparities persist in breast cancer treatment, communication in healthcare, long-term health outcomes and mortality [3, 4]. Women of lower socioeconomic status (SES) diagnosed with early stage breast cancer (I to IIIA) report significantly poorer communication with their clinicians, lower knowledge of breast cancer surgery options, higher uptake of mastectomy, and worse cancer-related and patient-centered health outcomes compared to women of higher SES [3–11]. They also tend to receive breast cancer care that deviates from established clinical guidelines (e.g., inconsistent use of radiation after breast conserving surgery) [4, 10].

SES-linked differences in early stage breast cancer care meet the Institute of Medicine’s definition of a health service disparity [3, 9, 12, 13]. For early stage breast cancer, lower SES is a stronger predictor of poor outcomes and treatment received than race or ethnicity [14, 15]. Treatment disparities are associated with patient-, clinician-, and system-level factors [16, 17]. The reasons women of lower SES are more likely to choose mastectomy over breast conserving surgery (BCS) are not well defined [8]. Limited financial resources and lack of insurance coverage do not always predict decision-making for early stage breast cancer surgery [18]. Moreover, BCS has higher short-term cost but lower long-term cost [19]. For many women of lower SES, Medicaid, Medicare, and other programs will cover the costs of either treatment, thus minimizing the impact of treatment cost on decision-making. Patient-level factors, such as childcare, transportation expenses, and financial pressures (e.g., a need to return to work quickly) may be strong influences on treatment choices as well. However, the exact impact of these factors remains unclear [20, 21].

Although BCS is a recommended treatment for early stage breast cancer, research confirms equivalent survival between mastectomy and BCS [22–25]. Both options are offered routinely yet have distinct harms and benefits that patients may value differently [26]. In this context, patient preferences play an essential role in decision-making. According to the Institute of Medicine, shared decision making (SDM) should be promoted to improve the quality of health care, particularly for cancer care [27–31]. However, only 44 to 51% of women with early stage breast cancer across socioeconomic strata achieve the degree of participation in decision-making they desire [5, 6, 32–35]. Women of lower SES are more likely to play a passive role in decision-making and to have higher decision regret following surgery than women of higher SES [4, 5, 8, 9, 11]. Surgeons may spend less time communicating and engaging with patients of lower SES [11, 36]. The relationship between SES, participation in decision-making, and breast cancer disparities is robust [3, 4, 7, 9, 10, 12].

Patient decision aids may help reduce those disparities by providing evidence-based information about the harms and benefits of options to help patients deliberate about their preferences [37]. Patient decision aids for breast cancer surgery reduce decisional conflict, increase knowledge and satisfaction with the decision-making process, and, in some instances, increase BCS uptake and quality of life [38–40]. However, decision aids are often designed for highly literate audiences, may have poor accessibility and/or readability, and may not be tailored to the needs of individuals of low SES and low health literacy [38, 39, 41–46]. All but one breast cancer decision aids were evaluated solely in women of higher SES [47]. Shorter, simpler decision aids designed for use in clinical encounters—encounter decision aids—may be more beneficial to underserved patients, provided they are developed to meet the needs of these patients [41, 42, 48]. Encounter decision aids can increase patients’ knowledge and participation in decision-making, improve risk perception, and, in some instances, influence choice and improve adherence to treatments [49–54]. They have been successfully used by clinicians, do not increase consultation time, and are becoming routinely adopted in usual care through the electronic medical record [51, 55–57]. However, the effect of encounter decision aids among patients of lower SES and lower health literacy, to potentially reduce disparities across socioeconomic strata, has never been evaluated.

**Fig. 1:**
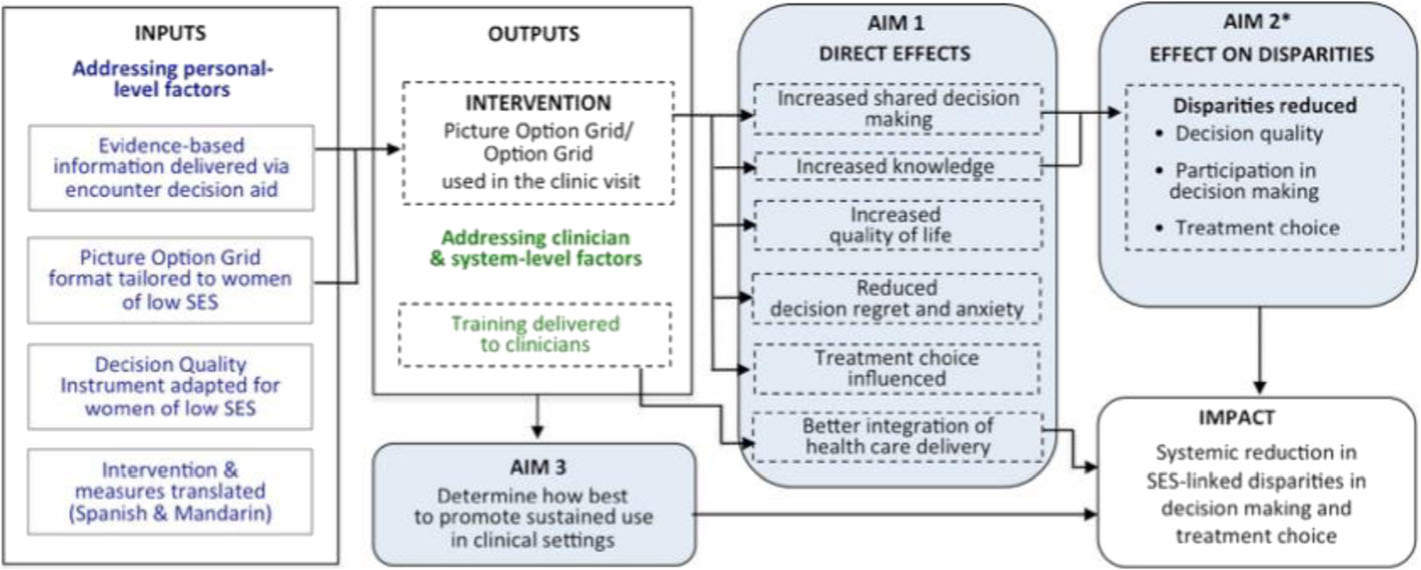
Logic model of proposed study. *See Fig. 4 for mediation pathways. Legend: blue text = Personal level factors according to Cooper’s framework, green text = Clinician & system level factors according to Cooper’s framework, - - - - outline = outputs and outcomes of the randomized controlled trial

The three aims of our study will be realized in the context of the logic model shown in Fig. 1.

### Aim 1

Assess the comparative effectiveness of two effective encounter decision aids (Option Grid and Picture Option Grid) against usual care on SDM, decision quality, treatment choice and other direct outcomes in women, and differentially by SES.

### Hypothesis 1.1

The encounter decision aids will increase SDM in the clinic visit and improve decision quality, knowledge, and quality of life in women of higher and lower SES compared to usual care. We also anticipate that they will reduce decision regret and improve the perceived integration of healthcare delivery (see Fig. 1).

### Hypothesis 1.2

The Picture Option Grid will be more effective than the Option Grid at improving primary and secondary outcomes in women of lower SES. There will be no difference between the effects of the two encounter decision aids in women of higher SES.

### Aim 2

Measure the effect of the Picture Option Grid on disparities in decision-making (decision quality, knowledge, and SDM) and treatment choice, and conduct an exploratory analysis of the mediation and moderation effects.

### Hypothesis 2.1

Compared to the Option Grid and usual care arms, the Picture Option Grid will reduce disparities in decision quality, knowledge, and participation in SDM between women of lower and higher SES. It is also likely to reduce disparities in treatment choice.

### Hypothesis 2.2

**Fig. 2:**
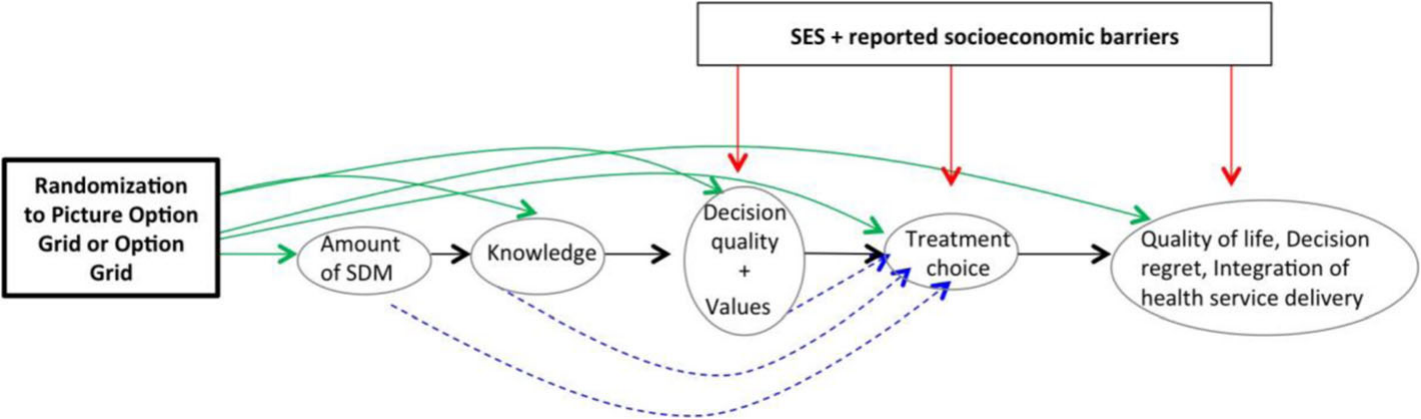
Causal model for patients enrolled in the trial. Legend: Arrows depicted in green, red and blue represent causal relationships of one variable on another. The presence of green arrows will be examined in Aim 1. The presence of blue arrows (mediation effects) and red arrows (moderation effects) will be examined in an exploratory analysis in Aim 2

The effect of the Picture Option Grid on treatment choice will be mediated by post-intervention knowledge, SDM, and post-intervention values (reported in ‘What Matters Most to You’ subscale of DQI, e.g., keeping breast, removing breast to gain peace of mind, avoiding radiation, etc.) (see Fig. 2).

### Hypothesis 2.3

For women of lower SES, socioeconomic barriers (e.g., resource constraints, as reported in the Decision Quality Instrument) will affect treatment choice and thereby moderate the intervention’s effect.

### Aim 3

Explore strategies that promote the encounter decision aids’ sustained use and dissemination using a theoretical implementation model.

### Hypothesis 3.1

Pre-visit planning, minimal clinician training, flexibility of use, and integration into the workflow and EMR will facilitate sustained use.

### Hypothesis 3.2

Successful use by patients and their families will be determined by the perceived acceptability of the intervention and integration into workflows.

## Methods

This clinical trial protocol follows the SPIRIT guidelines (see Additional file 1) and CONSORT statement [58, 59].

### Design

**Fig. 3:**
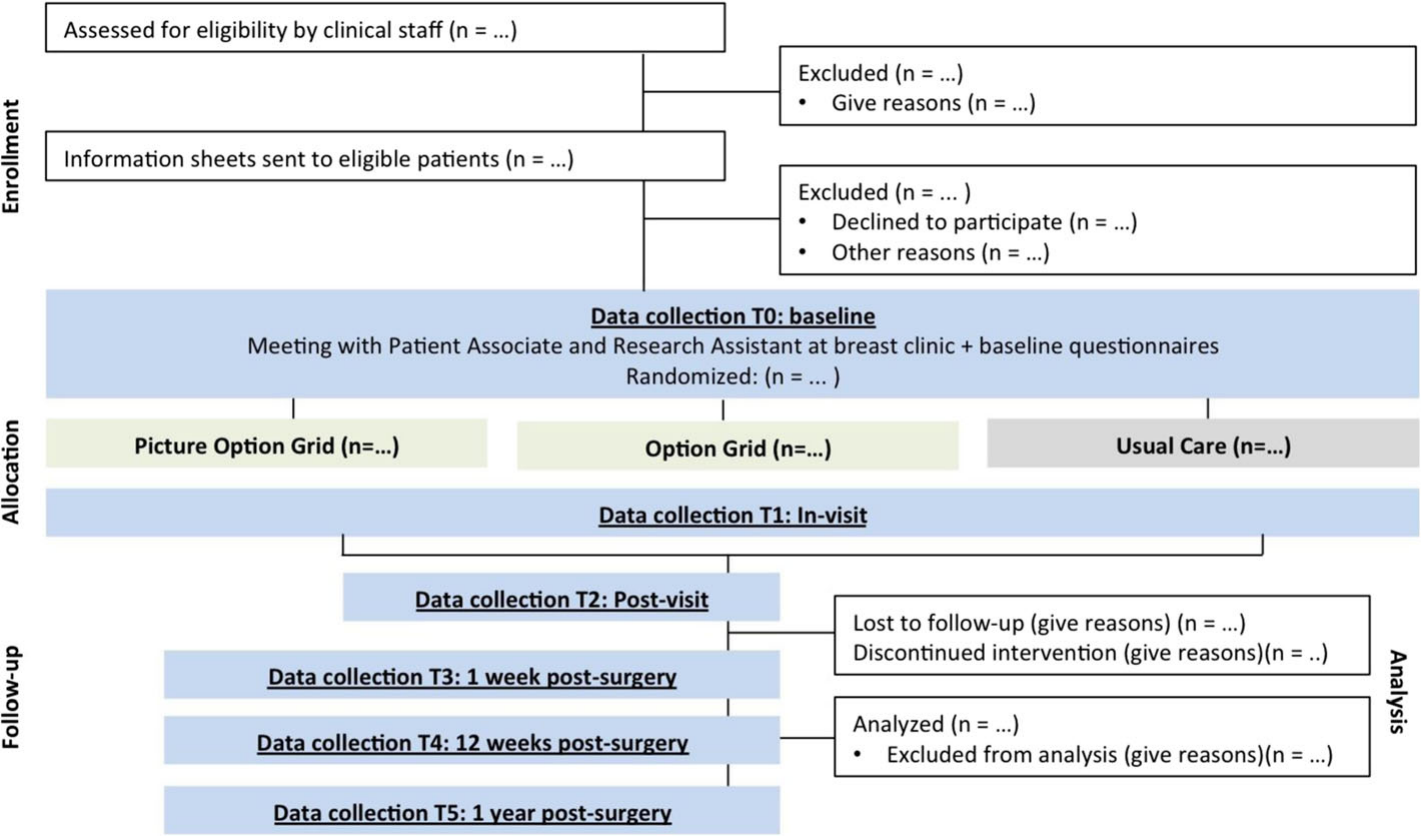
CONSORT* study flow diagram. *CONSORT stands for Consolidated Standards of Reporting Trials, as reported in the CONSORT statement

We will use a three-arm, multi-site randomized controlled superiority trial with stratification by SES and parallel study design. Over a 16-month period, we will recruit 1100 patients (half higher SES and half lower SES) (Fig. 3). Randomization will be at the clinician level, nested within study sites, and will involve data analyst blinding. At each site, we will use a cross-sectional study design and randomize participating clinicians to one of three arms (Option Grid, Picture Option Grid, or usual care) using an R script written by the study statistician. Balanced block randomization will be used to account for the varying number of physicians at each site. Patients who have given informed consent and are seeing one of the participating clinicians will be allocated to the clinician’s corresponding arm. In Year 1, we will adapt the “What Matters Most to You” subscale of the Decision Quality Instrument (DQI) for women of lower SES. For Aim 3, we will use interviews with trial participants, healthcare professionals, and other relevant stakeholders, field-notes, and observations to explore strategies that promote the encounter decision aids’ sustained use and dissemination. Community-Based Participatory Research (CBPR) will be used throughout the trial.

#### Controlling for contamination

Since the randomization occurs at the clinician level, we are confident that the risk of contamination will be minimal [60]. However, to control for any potential contamination and assess the fidelity of delivering each intervention, we will audio-record clinical encounters with patients who have consented to have their consultation recorded (see consent process in section Data management and analysis). At T2, we will ask all patients to indicate which intervention was introduced to them. We will use both intention-to-treat and as-treated analyses. We will train all clinicians in delivering the intervention according to their allocated arm (see section Monitoring for clinician training). Clinicians in usual care will not be trained in the use of the interventions. If there is residual contamination, the bias will be towards a null effect. We will therefore be confident that any significant findings are actual.

### Setting

In order to ensure that study participants are representative of the target population, the study will be conducted at four large cancer centers in the United States located in geographically diverse regions that provide a combination of urban and rural settings as well as racially and ethnically diverse populations (in the full trial protocol, available upon request).

### Participants

#### Stratification by SES

Insurance status will be used to screen for higher and lower SES (lower SES: uninsured, on Medicaid or Medicare without supplemental insurance or ACA Marketplace plans; higher SES: privately insured or on Medicare with supplemental insurance). At baseline, we will collect information about median household income, household size, and highest educational attainment (self-reported). We will use household income and highest educational attainment to measure SES, in combination with insurance status (using multiple informants analysis). This approach is acceptable and recommended by experts [61–63].

#### Randomized controlled superiority trial (aims 1 and 2)

We will recruit 1100 women at least 18 years of age with a confirmed diagnosis of early stage breast cancer (I to IIIA) over 16 months. Approximately half will be from the lower SES population, and about half will be from the higher SES population.

**Participant Inclusion Criteria**

- Assigned female at birth;
- At least 18 years of age;
- Confirmed diagnosis (via biopsy) of early stage breast cancer (I-IIIA);
- Eligible for both breast-conserving surgery and mastectomy based on medical records and clinician’s opinion before surgery;
- Spoken English, Spanish, or Mandarin Chinese.

**Participant Exclusion Criteria**

- Transgender men and women;
- Women who have undergone prophylactic mastectomy;
- Women with visual impairment who might have difficulty viewing the decision aids;
- Women with a diagnosis of severe mental illness or severe dementia;
- Women with inflammatory breast carcinoma.

Women who are receiving neoadjuvant therapy will be invited to participate in the first 9 months of the trial in order to complete follow-up assessments before the trial terminates. T3 assessments will occur after neoadjuvant therapy and post-surgery. This may occur up to 7 months after T0 (baseline).

#### Feasibility of recruitment

We aim to recruit 275 patients at each site (this number may vary depending on stratification and patient volume). To facilitate recruitment, a patient associate will be employed at each site throughout the study. The patient associate will be a patient who has had breast cancer and has completed all treatments. She will be involved in the trial to promote patient-centeredness and facilitate recruitment among women of lower SES. The patient associate will be CITI certified and trained to consent and recruit participants. The Institutional Review Boards have no concerns about the involvement of a patient associate who has completed all breast cancer treatments (see section Tracking and retaining participants for tracking and retention of participants).

### Interventions and comparators

The interventions are paper-based and range from one to four pages in length. The Option Grid was written in plain language but was not specifically designed for women of lower SES (Flesh-Kincaid grade level of 6.6). The Picture Option Grid (specifically designed for women of lower SES and lower health literacy) has a Flesh-Kincaid grade level of 6.5. Both interventions will be used by the surgeon during the surgical consultation. By using the same medium and delivery mode, we enable a direct comparison. Both interventions have been developed, tested, and validated [51, 64–68].

#### Intervention 1: Option grid

The Option Grid™ encounter decision aid for early stage breast cancer surgery is a one-page, evidence-based summary of available options in a tabular format (Additional file 2). The efficacy of Option Grid decision aids has been tested in a stepped wedge trial, where they were shown to increase patients’ knowledge and SDM in the clinic visit [51]. Similar results were achieved using qualitative methods [55, 57, 69, 70]. Option Grid decision aids are used in routine clinical practice and downloaded over 5000 times a month (http://www.optiongrid.org/). The Option Grid for breast cancer surgery was developed in 2010 and downloaded 1346 times in 2016. It was initially adapted from a web-based decision aid shown to facilitate readiness to decide and strengthen surgery intentions [67, 68].

#### Intervention 2: Picture option grid

The Picture Option Grid was derived from the Option Grid for early stage breast cancer (see Additional file 3). It uses the same evidence but integrates images and simpler text in order to exploit picture superiority [71, 72]. The Picture Option Grid has been specifically designed for women of lower SES and lower health literacy. It was iteratively developed and tested in underserved community settings with lay women (without breast cancer) and breast cancer patients of lower SES using CBPR [66]. We have tested its acceptability, feasibility, and perceived impact in 278 women of lower SES diagnosed with early stage breast cancer and with health professionals, comparing it to the Option Grid and to a comic strip encounter decision aid [65]. Most women of lower SES and health professionals deemed the Picture Option Grid most acceptable and usable.

#### Comparator

Because decision aids are not routinely available in clinical settings, usual care is a legitimate comparator. For the purpose of this trial, usual care will include the provision of typical information resources about breast cancer that are currently available at each study site. These resources differ across study sites. To capture differences, we will collect detailed information about usual care at each site using methods derived from ethnography and will include questions about usual care at T2.

### Outcomes

To accommodate varying levels of literacy and health literacy of our target group, we will use validated short-form questionnaires whenever possible. Aside from the Observer OPTION^5^ scale [73], all are patient-reported outcome (PRO) or patient-reported experience (PRE) measures. All included scales and tests other than the demographic questions have been validated.

#### Primary outcome measure

The primary outcome measure (see Table 1) is the validated 16-item Decision Quality Instrument (DQI) for breast cancer, which includes a knowledge subscale [74] (in the full trial protocol, available upon request). The DQI, designed to be administered post-decision, will be assessed at T2 and T3. It aims to measure the extent to which patients are informed about their options, are involved in the decision-making process, and receive a surgery (mastectomy or BCS) aligned with their values, attitude towards risks, and preferences. It produces three scores: (1) knowledge, (2) concordance, and (3) decision process [74].

**Table 1:** Outcome measures according to data collection periods

	TIMEPOINT						
	-T0	T0 Baseline	T1 In-Visit	T2 Post-Visit	T3 1 wk. PS^a^	T4 12 wks PS^b^	T5 1 yr. PS
CONSENT AND ENROLLMENT							
Eligibility Screen	X						
Informed Consent	X						
Allocation (via surgeon confirmation)	X						
INTERVENTIONS							
Arm 1: Option Grid			X				
Arm 2: Picture Option Grid			X				
Arm 3: Usual Care			X				
OUTCOME MEASURES							
Rates of recruitment – documented and tracked in REDCap		X					
Discontinuation rates – documented and tracked in REDCap		X		X	X	X	X
Demographic data – 6 items, self-reported		X					
Health literacy – 1-item Chew’s health literacy screening		X					
Decision quality (primary outcome measure) – validated 16-item DQI, subscale adapted for low SES				X	X		
Knowledge – validated 5-item DQI knowledge subscale		X		(X)	(X)		
Treatment intention – self-reported via DQI				(X)			
Treatment choice – obtained from medical records					X		
Quality of life – validated 6-item EQ-5D-5 L		X				X	
Anxiety – validated 8-item PROMIS anxiety short form		X		X	X	X	
Shared decision-making (observed) – validated OPTION^5^			X				
Shared decision-making (self-reported) – validated 3-item CollaboRATE				X			
Decision regret – validated 5-item decision regret scale					X	X	X
Integration of health care delivery – validated 4-item IntegRATE		X				X	
Financial toxicity – four items from validated COST measure and self-report of out-of-pocket medical expenses in the past month					X	X	X
Intervention’s patterns of use – questions and photos of intervention				X	X		
System level factors + feasibility and acceptability in routine care							
Ethnographic methods		X	X	X	X	X	
Semi-structured interviews						X	

*PS* post-surgery

(X) included in full DQI

^a^or first post-operative visit

^b^or second post-operative visit

#### Secondary outcome measures

Secondary outcome measures will include treatment choice (assessed at T3 using patients’ medical records), treatment intention, CollaboRATE (the validated three-item measure of SDM) [75, 76], Chew’s validated one-item health literacy screening question [77], PROMIS, an eight-item validated short form measure of anxiety [78], EQ-5D-5 L, the validated, standardized six-item quality of life measure [79], the five-item validated decision regret scale [80], and four items from COST, a validated financial toxicity measure [81, 82]. We will also ask participants to estimate the out-of-pocket (OOP) portion of their medical expenses over the past month. We will use the recordings of clinical encounters (see section Controlling for Contamination) to analyze the extent to which SDM occurs using the five-item validated observer-rated OPTION^5^ scale [73]. We will include a fidelity-of-use checklist derived from Wyatt’s work to assess the actual use of encounter decision aids [48]. Finally, we will use IntegRATE, a four-item generic patient-reported measure of integration of healthcare delivery [83]. At T2 and T3, we will investigate each intervention’s patterns of use. At T2, the research team will take a picture of each intervention post-consultation to determine how the intervention has been used and whether the patient/family and/or clinician have annotated it. At T3, participants will be asked to indicate how many times they have used the intervention and whether family members, relatives, or caregivers have used the intervention.

### Translation procedures

All study documents, interventions, and measures that are not currently available in Spanish and Mandarin Chinese will be translated using standard translation procedures successfully implemented before [84]. Spanish and Mandarin Chinese interpreters or ‘language lines’ will be available at each site before, during, and after the clinic visit.

### Sample size and power calculation

For Aim 1, hypothesis 1, we base the effect size estimation on published data from randomized controlled trials of decision aids for breast cancer surgery [38, 39, 85, 86], suggesting that a reasonable effect size for DQI is 9.34, that the standard deviation between patients in the intervention arms compared to usual care is 12.00, and that a within-physician intra-class correlation (ICC) of 0.05 is reasonable. This ICC is justified because treatment varies within study site, thereby allowing heterogeneity that occurs between physicians across centers to be blocked. We assume a patient attrition rate of 20%. Under these assumptions, a study of 1100 participants (68.75 participants per physicians and 366.66 per treatment group) has power of greater than 99.8% to reject the null hypothesis that the encounter decision aid groups and the control group have equal means, using a two-sided 0.05 level test when the true mean difference is 9.34. For hypothesis 2, because we anticipate obtaining a similar number of women in the higher and lower SES categories, the same power calculation is performed on a sample size of half the size. With 550 patients in total (34.375 patients on average per physician), under the same assumptions as above, the power for this subgroup test is 99% with ICC = 0.05 and with ICC = 0.175.

For Aim 2, the power of the test for disparities between higher and lower SES is necessarily lower than the overall test at the same effect size, as four groups are compared (Picture Option Grid higher SES, Picture Option Grid lower SES, Option Grid and usual care higher SES, Option Grid and usual care lower SES). However, because patient SES varies within physician, power can be conservatively computed as if the patient-level variance was doubled and the total number of patients halved. If the true difference in the effect of the Picture Option Grid on decision quality between the higher and lower SES groups is 8.5 compared to Option Grid or compared to usual care, the power for a two-sided alpha-level test at the 0.05-level is just above 80%. A significant finding is even more likely to be obtained if the estimated variability in the data turns out to be much smaller than assumed here. It is not critical to account for multiple testing because in both the primary (Aim 1) and secondary (Aim 2) analyses, a single pair of groups is compared.

### Screening, consent, and allocation

#### Screening for inclusion in the randomized controlled trial

At each study site, our research assistants will work with the breast care team to identify eligible participants in advance of each breast clinic. Insurance status (self-reported or obtained via EMR) will be used to screen for SES. For each participant, we will check whether her income (in EMR or self-reported at baseline) is consistent with the higher or lower SES designation made at the initial screening, and reassign if needed. As mentioned above, educational attainment will also be collected but will not be used during the recruitment process to determine allocation to the higher or lower SES group.

#### Consent procedures

**Fig. 4:**
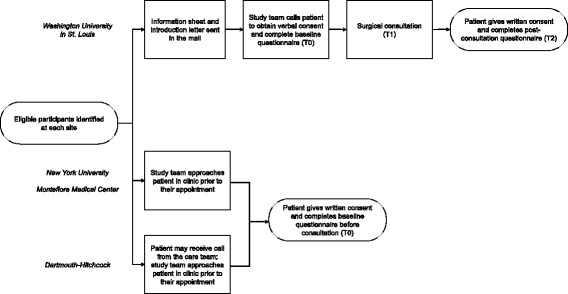
Site-specific consent and baseline assessment procedures

Eligible patients at Washington University in St. Louis will receive an information sheet a few days before their scheduled appointment. The information sheet will be written using plain language and pictures to address the needs of women of lower SES and low literacy/low health literacy. At NYU School of Medicine, Montefiore Medical Center, and Dartmouth-Hitchcock, the research assistant will approach patients who are pre-screened for eligibility on the clinic day to provide the information sheet and discuss the study prior to the surgical appointment. Consent procedures will vary slightly at each site based on the patient flow and individual needs of each clinic. However, at each site, the research assistant will be responsible for obtaining written consent for participation in the trial (including recording of clinical encounters and interviews). The research assistant or patient associate will read the questions to patients who cannot read or write by using standardized interviewing procedures in a private room. We will document whether a standardized interview was conducted and whether the assistance of an interpreter was needed (see Fig. 4 and full trial protocol available upon request).

#### Assignment of interventions

Patients who have given informed consent and are seeing one of the participating clinicians will be allocated to their clinician’s corresponding arm (Option Grid, Picture Option Grid, or usual care). The intervention will be used during the surgical consultation visit. In the usual care condition, the patients will receive care as usual for their respective study site. They will receive a study card at the end of the consultation to signal to the research team that the patient has been officially enrolled into the study.

We will monitor accrual at each site to ensure that a similar number of patients is recruited in each socioeconomic stratum and arm. Once the target number of participants in each arm and each stratum has been reached, recruitment of patients in this particular arm and stratum will end. Recruitment will continue in other arms and strata until all target numbers of participants per group have been reached.

#### Changes to intervention allocation

There are no established criteria for discontinuing or modifying the allocated intervention for study participants due to the low risk nature of the trial. If a participant requests to be put in a different arm, they will not be able to take part in the study. Subjects will be free to withdraw from the study at any time for any reason.

#### Baseline and follow-up assessments

The baseline assessment will happen prior to the first surgical consultation visit. Follow-up assessments will occur immediately after the surgical consultation (same day), 1 week post-surgery, or at the first post-surgical clinic visit (T3), 12 weeks post-surgery, or at the corresponding clinic visit (T4), and 1 year post-surgery (T5). Baseline and follow-up assessments will be completed by the research assistant or patient associate. They will be done in person, online, over the phone using standardized interview procedures, or via mail service.

### Data management and analysis

#### Data management

Data management for the study will be done through REDCap, a HIPAA-compliant web-based data management system [87]. REDCap will be hosted at Dartmouth-Hitchcock Medical Center (DHMC) for data collected at DHMC. Data collected from all other sites will be hosted on REDCap at Dartmouth College. Access will only be granted to study team members designated to manage the study data and will require a dedicated username and password. Study team members at participating study sites will only have access to data collected at their institution. This database management system is designed to comply with the ICH Good Clinical Practice (GCP) guidelines.

Data entry into REDCap will be done by research assistants and patient associates at each site using standardized data collection forms. Samples of the data collection forms will be available in the full protocol once they are finalized (available upon request).

In addition, each study site will have a data-protected, encrypted external hard drive for the local storage of sensitive study-related materials. Each of the study sites will return the hard drives to Dartmouth College at the end of the trial. Dartmouth College will store the encrypted data for 6 years after the conclusion of the trial, after which all data will be destroyed. Signed consent forms will be kept in a locked file cabinet in a secure location at each study site, and for 6 years after the conclusion of the trial.

#### Data analysis

Initial examination of data will include descriptive statistics, frequency distributions, and histograms in order to identify outliers and missing data. Primary analyses will be based on “intention-to-treat”, but an “as-treated” analysis based on participants’ report of the intervention received, will also be undertaken.

##### Analysis corresponding to aim 1

We will first perform separate analyses for each follow-up period using linear and logistic regression models as appropriate for continuous (decision quality, SDM, quality of life, anxiety, decision regret, and IntegRATE) and binary (treatment choice) outcomes respectively. The results will provide potentially valuable insights into how rapidly each intervention affects outcomes. Outcomes measured multiple times after T0 (anxiety, regret, decision quality, and financial toxicity) may also be analyzed using a longitudinal model. If the interventions are found to have an effect, a secondary analysis that adds predictors for the number of prior Option Grid and Picture Option Grid patients seen by the healthcare professional will examine whether there are physician learning effects under either intervention.

We will adopt a regression framework for all analyses as it allows seamless transition between basic analyses involving a single predictor (or two indicators corresponding to each intervention versus the comparator) and more complex analyses involving additional predictors (mediation variables, control covariates, time-trends, interaction terms or effect modifiers). Further, the regression framework allows clustering of observations due to repeated measurements on patients across time, nesting of health professionals within sites, and patients within health professionals, to be accurately accounted for using mixed-effect regression models [88] or generalized estimating equations [89, 90]. Multiple comparisons will be accounted for using Scheffe’s method [91].

The secondary outcomes (SDM, anxiety, integration of healthcare delivery, decision regret, quality of life and financial toxicity) have in excess of 10 levels, and they will be analyzed as continuous variables. To assess whether the results of each analysis are trustworthy, we will analyze the residuals to check if the assumptions of the model hold [92]. For treatment choice, a clearly defined binary variable based on medical record data, we will adapt the model to a logistic regression model.

The three measures of baseline socioeconomic status: (1) insurance status, (2) highest educational attainment, and (3) median household income, will be analyzed separately for a multiple informants analysis [93] or, provided they are not excessively collinear, we will enter them in the model together and test their combined effect. For income, we will use a poverty income ratio: the ratio of household income accounting for household size and poverty line published by the Census Bureau in that calendar year [94]. To aid interpretation of our results, we will report the consequence of a patient changing from above median SES to below median SES, even if for added precision, it makes sense to base significance tests on continuous measures.

To gain insight into whether the Picture Option Grid and Option Grid will be more effective in certain subpopulations, we will add each SES measure and its interaction with the intervention indicator variables to the model. If the SES intervention interaction is non-significant, we will remove them from the model and test if the overall effect of SES is significant. Otherwise, we will perform stratified analyses of the interventions’ effects by SES status.

##### Analyses corresponding to aim 2

A logistic regression model will be used to test for differences between the Picture Option Grid group and the usual care and Option Grid groups in decision quality, knowledge, participation in SDM, and treatment choice, within subpopulations (higher SES versus lower SES). A reduction of disparity due to the interventions will be claimed if the effect of SES on outcomes is significantly smaller for the Picture Option Grid group than for the other two groups at follow-up. As for Aim 1, a linear regression model will be used for the decision quality analysis while an analogous set of other predictors will be included as covariates in the model. The assumptions of the models will be evaluated for adequacy using residual analysis and other model fit diagnostics [92]. Our exploratory mediation analyses seek to identify and explicate the mechanism or process that underlies the relationship between the Picture Option Grid and a dependent variable via the inclusion of a third explanatory variable, known as a mediator variable (e.g., knowledge, values, SDM). We are specifically interested in whether interventions operate through the mediator as opposed to directly affecting the outcome. We will perform these analyses even if the findings from Aim 1 and Aim 2 (hypothesis 1) are non-significant in order to determine whether the null effect was due to a null effect of the intervention on the mediator or a null effect of the mediator on the outcome. To determine the generalizability of these mechanisms and identify subpopulations for whom mediation is most pronounced, we will compare the mediation effects across different subgroups (e.g., higher SES versus lower SES).

The traditional and often-used approach to estimating mediation effects is the causal steps approach, which originated in Baron and Kenny (1986) [95]. However, due to the limitations of that approach, we will estimate the mediation effect using the product of coefficients method [96]. Standard errors will be evaluated using the bootstrap [97] or the PRODCLIN program [98]. Software exists for sensitivity analysis to violations of sequential ignorability and other assumptions required for causality in mediation analyses [99, 100]. We will apply this software and any additional procedures available to our analyses in order to obtain the most robust and defendable results.

##### Analyses corresponding to aim 3

We will use a framework analysis, guided by Normalization Process Theory (NPT) [101, 102], having successfully used this approach previously [55, 70, 103, 104]. Observations and field-notes will be included in the analysis. Initial descriptive codes will be generated by two independent researchers based on the four NPT constructs. In-vivo coding will also be used to capture other naturally occurring exchanges. Categorical codes that group initial and in-vivo codes will be developed in a third round of coding. In addition, 100 photos of the interventions taken at T2 (approximately 50 of Option Grid and 50 of Picture Option Grid) will be included in the analysis to answer questions 1 and 2. Triangulation of data will also be performed.

NPT was developed to understand how complex interventions become implemented in routine healthcare settings [101, 102]. It is built around four theoretical constructs: 1) Sense-making: processes of individual and communal sense-making of a complex intervention regarding its use and value; 2) Participation: processes of ‘cognitive participation’ that promote or hinder users’ buy-in and commitment to the intervention; 3) Action: processes of ‘collective action’ that determine or hinder whether the intervention is being used by all as intended; and 4) Monitoring: Processes of communal and individual appraisal of the effect of the intervention. We will use NPT as an analytical lens to consider the data collected according to our hypotheses and the following five questions: (1) how the interventions were perceived and used in and outside the clinical encounter (including with family and caregivers), (2) preferred ways for introducing and using the intervention in routine clinical settings, from several perspectives: patients, family, health professionals, administrators (3) perceived fit in clinic workflow as well as reported barriers and facilitators to routine integration, (4) other perceived patient-, physician- and system-level barriers and facilitators to routine use, and (5) perceived generalizability and feasibility in routine care.

### Missing data

Most data collection will be via questionnaires (at T0, T2, T3, T4, and T5), which provide opportunities for preventing and monitoring missing data. We will offer other formats for questionnaire completion (including standardized interviews), thus minimizing missing data. We will prompt each patient, by telephone, to complete the follow-up questionnaires (at T3, T4, and T5) or reach them in the clinic during their post-surgery appointments. Given the brevity of the trial and the procedures described above, we do not anticipate more than 5% of missing data. However, should there be more than a trivial amount of missing data, we will use multiple imputation to cope with missing baseline, interim, and outcome data (105). We will record and report all reasons for dropout and missing data. This approach will address both generalizability and causal validity bias. We will also examine sensitivity of inferences.

#### Heterogeneity of treatment effects

The main goals of the heterogeneity of treatment effects (HTE) analyses are to estimate treatment effects in clinically relevant subgroups and to predict whether an individual might benefit from exposure to the decision aid. As the HTE analyses are exploratory rather than hypothesis-driven, exploratory subgroup analyses will be conducted to identify hypotheses for future evaluation. Patient characteristics will be considered for treatment by covariate interactions and include SES, age, ethnicity, race, literacy, language, and study site [105]. As described in the analytic plans for testing interactions by SES in Aims 1 and 2, interaction tests will be conducted to determine if subgroup analyses of the intervention effects by the levels of that predictor are warranted. If the interaction is significant, then the treatment effect is estimated separately at each level of the categorical variable used to define mutually exclusive subgroups.

#### Access to complete dataset

Only the statistician and core research team will have access to the final data set.

All data used in conducting the final analyses will be made available in a de-identified copy for archival purposes, and for collaborating researchers and organizations in no more than 9 months from the end of the final analysis. We also plan to develop a Data Access, Analysis, and Expression of Interest submission and review process for formal requests to make use of the data, thus preventing duplication in analysis and publication. Given the data sharing plans, we will provide a detailed description of these plans to all participants during the informed consent process to ensure that participants are aware of all potential uses of data.

### Clinician training

All participating clinicians will receive training in using the intervention they are randomized to, as well as how to adhere to the trial protocol. This will include basic shared decision making and communication skills training. We will use videos and role-plays. For clinicians who are not able to attend a training session in person, training will be done on the phone. A video will also be available.

### Monitoring

#### Monitoring enrollment

Enrollment will be monitored weekly at each site. The number of patients screened by the breast-care team, proportion eligible (and sent an information sheet), and proportion consented and recruited in-clinic (according to SES strata) will be documented on a screening log. Where possible, reason for dropout will be recorded.

#### Adherence to protocol and supervision

To maximize adherence to the trial protocol, we will train all co-investigators, research assistants, and patient associates in recruiting patients according to the procedures outlined in the protocol. The protocol will be made available to all research team members and key stakeholders in a password-protected section of the website. Feedback will be provided to participating surgeons and other members of the study team at each study site as necessary, at 3 months, 6 months, and 12 months into recruitment. To provide feedback to participating surgeons, we will use a preliminary analysis of the audio recordings of randomly selected consultations across all three arms as well as field-notes. Any proposed changes will be discussed with the Trial Steering Group then reported to PCORI, the Data and Safety Monitoring Board (DSMB), IRB, and noted on ClinicalTrials.gov.

#### Trial management

Dartmouth College will have responsibility for centralized study management and general oversight. The research team at Dartmouth will maintain all aspects of the trial and work closely with each study site to coordinate all trial activities.

#### Trial steering group

A Trial Steering Group (TSG) will involve all key personnel (including patient and stakeholder partners and invited CAB members) and will meet every 3 months. The duties of the TSG will include supervising the trial, monitoring trial progress, as well as reviewing and acting on all DSMB recommendations.

#### Data and safety monitoring board

A Data and Safety Monitoring Board (DSMB) will be appointed to provide additional oversight in the trial. DSMB membership will comprise of seven members including experts in the fields of shared decision making, breast cancer surgery, patient advocacy, statistics, and clinical trials methodology. The DSMB will operate independently from the study sponsor. The DSMB charter is available in the full trial protocol (available upon request). The DSMB will meet and review data bi-annually throughout the project. The DSMB will review the protocol, data collected to date (interim analyses every 6 months), and advise the PI on any potential risks and risk mitigation plans. We do not expect any Serious Adverse Events (SAE) or Adverse Events (AE) that would require immediate reporting. However, some patients, particularly those with diagnosed mental illness or patients who are finding it difficult to cope with their recent cancer diagnosis, may find it stressful to be randomized to one of the study arms. The DSMB will therefore review data on subject withdrawals from the study and stated reasons for withdrawal as well as study subject anxiety scores (measured using PROMIS) for each withdrawal. A detailed plan for identifying and reporting participants who may be experiencing heightened anxiety leading to withdrawal is provided in the full protocol (see supplemental file full trial protocol). If for any reason, an SAE or AE were reported, the IRB at Dartmouth College would be immediately notified as well as the appropriate safety board at the participating sites. The DSMB would convene urgently and review the SAE/AE.

### Tracking and retaining participants

The research assistant or patient associate employed at each site will track participants and ensure that they are called in advance of the follow-up assessments and provided with a questionnaire in the format of their choice. Interpreter services will be used whenever necessary. Telephone calls will also be made by the patient associates whenever they are available.

Retention among patients of lower SES will be maximized by:

- Using short-form validated measures;
- Translating those measures into Spanish and simplified Mandarin Chinese;
- Using interpreter services whenever necessary;
- Calling all participants 2-5 days before each follow-up assessment is due, prompting patients to complete the questionnaires and offering to conduct a standardized interview over the phone. The latter is likely to be particularly helpful in patients of lower SES and lower literacy/health literacy;
- Conducting follow-up assessments in-person when possible;
- Giving patients a choice of questionnaire format for the completion of baseline and follow-up assessments (online, paper-based, or standardized interviews);
- Compensating participants for their time. Brueton et al. identified monetary incentives as an effective way of improving participant retention [106].

### Ethics and dissemination

Ethical approval has been sought for Montefiore Medical Center and Dartmouth-Hitchcock through Dartmouth College’s Committee for the Protection of Human Subjects (CPHS) (ref: STUDY00030157). Dartmouth College CPHS approved the study on June 8, 2017. Montefiore Medical Center Institutional Review Board (IRB) provided authorization agreement to rely on review by Dartmouth College on September 8, 2017. Ethical approval for Washington University in St. Louis was provided by The Washington University in St. Louis IRB on May 9, 2017 (ref: 201,704,011). NYU School of Medicine IRB provided ethical approval on August 29, 2017 (ref: i17-00871).

The study outputs will likely interest a wide variety of target audiences, ranging from patient and advocacy groups, healthcare professionals, and healthcare organizations to academics, policy makers, and decision aid developers. Since the interventions are easily accessible and inexpensive to update and disseminate, implementation in routine care could occur immediately post-project completion. We plan to disseminate findings through the following channels: academic, patient and advocacy organizations, professional organizations and healthcare delivery systems, social media and lay press, and dissemination symposia and clinician training modules (see more information in full trial protocol supplementary file). For academic outputs, we will follow ICMJE authorship guidelines.

## Discussion

Our study addresses an important research and implementation gap by evaluating two strategies for engaging all patients, and particularly those of lower SES, to reduce disparities. If this study shows the advantage of the Option Grid and/or Picture Option Grid, dissemination of those interventions could improve decision quality, knowledge, quality of life, and other outcomes while promoting informed treatment choice irrespective of SES and health literacy, compared to usual care.

We anticipate that findings could be reproduced and the intervention(s) adopted by clinicians in clinical practice to rapidly improve delivery of care. We will investigate in Aim 3 how to facilitate this process, address potential obstacles to routine use, and support clinicians in implementing the intervention(s). We anticipate that the study outcomes have the potential to change the way clinicians inform and support patients in making breast cancer surgery decisions. The study findings will be beneficial to clinicians, policy makers and other national and community stakeholders who aim to engage underserved patients to improve outcomes across socioeconomic strata to reduce disparities. The findings will directly benefit patients, their families and caregivers, as well as inform academics and decision aid developers who strive to produce interventions that are beneficial to all and can be effectively implemented in routine care.

### Practical or operational issues involved in performing the study

First, if the interventions do not show all hypothesized effects but demonstrate, at the minimum, an effect on SDM and knowledge (expected, based on preliminary data), we will undertake Aim 2 and focus on the mediation analysis and impact on disparities in knowledge and decision-making. Second, we will address potential imbalance in SES by monitoring SES accrual at each site, for both control and intervention groups. Third, given the expansion of the Affordable Care Act, insurance status might not always be an accurate proxy for SES. To solve this problem, we will check whether each participant’s income and education are consistent with the high or low SES group designation made at the initial screening**.** Fourth, implementing regular supervision and providing feedback will maximize fidelity of decision aid use. Based on our experience of recording clinic visits, we are confident that recording 10% of all visits (*n* = 100) is realistic and feasible. Finally, regarding Aim 3, if the interventions do not demonstrate the hypothesized effects and implementation is not immediately warranted, we will explore barriers to the intervention’s success, at the patient, clinician, and system levels.

## Additional files

Additional file 1: SPIRIT Statement checklist- What Matters Most Protocol. (PDF 179 kb)

Additional file 2: Option Grid for early stage breast cancer. (PDF 237 kb)

Additional file 3: Picture Option Grid for early stage breast cancer. (PDF 1894 kb)

## Abbreviations

BCS: Breast-conserving surgery
CBPR: Community-Based participatory research
CITI: Collaborative institutional training initiative
DQI: Decision quality instrument
DSMB: Data & safety monitoring board
EMR: Electronic medical record
MO: Missouri
NH: New Hampshire
NY: New York
NYU: New York University
OOP: Out-of-Pocket
PCORI: Patient centered outcomes research institute
PI: Principal investigator
PRE: Patient reported experience
PRO: Patient reported outcome
REDCap: Research electronic data capture
SDM: Shared decision-making
SES: Socioeconomic status
