# Supplementary material for: What matters most: protocol for a randomized controlled trial of breast cancer surgery encounter decision aids across socioeconomic strata
Source: BMC Public Health. 2018 Feb 13;18:241. doi: 10.1186/s12889-018-5109-2 (PMC5812033; doi:10.1186/s12889-018-5109-2)
Supplement: Supplementary file 2 — Option Grid for early stage breast cancer. (PDF 237 kb) [file 12889_2018_5109_MOESM2_ESM.pdf]

Breast cancer: surgical options

Use this **Option Grid™** decision aid to help you and your healthcare professional talk about how to best treat your breast cancer. This decision aid is for women with early stage breast cancer (stages I to IIIA).

Frequently asked questions	Lumpectomy with radiation	Mastectomy
**What is removed?**	The cancer lump is removed, with some surrounding tissue.	The whole breast is removed.
**Which surgery is best for long-term survival?**	Long-term survival rates are the same for both surgeries.	Long-term survival rates are the same for both surgeries.
**What are the chances of cancer coming back in the breast?**	Breast cancer will come back in the breast in about 5 to 10 in 100 women (5-10%) in the 10 years after a lumpectomy.	Breast cancer will come back in the area of the scar in about 5 to 10 in 100 women (5-10%) in the 10 years after a mastectomy.
**Will I need more than one surgery?**	Possibly, 20 in 100 women (20%) may need another surgery to remove breast tissue or lymph node that have cancer.	Possibly, if your lymph nodes have cancer.<br>Yes, if you choose breast reconstruction.
**How long will it take to recover?**	Most women are home within 24 hours of surgery.	Most women are home within 24 hours of surgery. It may take longer with reconstruction.
**Will I need radiation after surgery?**	Yes, for up to seven weeks after surgery.	Radiation is not usually given after mastectomy.
**Will my lymph nodes be removed?**	If cancer has spread to the lymph nodes under your arm, your doctor will discuss with you whether you need more treatment such as surgery or radiotherapy.	If cancer has spread to the lymph nodes under your arm, your doctor will discuss with you whether you need more treatment such as surgery or radiotherapy.
**Will I need chemotherapy?**	You may be offered chemotherapy, but this does not depend on the surgery you choose.	You may be offered chemotherapy, but this does not depend on the surgery you choose.
**Will I lose my hair?**	Hair loss is common after chemotherapy.	Hair loss is common after chemotherapy.

Editors: Marie-Anne Durand (Lead Editor), Glyn Elwyn, Lisa Caldon, Kari Rosenkranz, Dale Collins Vidal, Stephanie Sivell, Malcolm Reed
Evidence document: http://optiongrid.org/admin/resources/grid/evidences/9.pdf?x=WBZ1nd4YE
This Option Grid does not constitute medical advice, diagnosis, or treatment. See Terms of Use and Privacy Policy at www.optiongrid.org.
Publication date: 23 August 2017 Expiry date: 23 August 2019
