# Supplementary material for: What matters most: protocol for a randomized controlled trial of breast cancer surgery encounter decision aids across socioeconomic strata
Source: BMC Public Health. 2018 Feb 13;18:241. doi: 10.1186/s12889-018-5109-2 (PMC5812033; doi:10.1186/s12889-018-5109-2)
Supplement: Supplementary file 3 — Picture Option Grid for early stage breast cancer. (PDF 1894 kb) [file 12889_2018_5109_MOESM3_ESM.pdf]

Early stage breast cancer: What's right for me?

Use this **Picture Option Grid** to help you and your healthcare professional decide how best to treat early stage breast cancer (stages I to IIIA). The last page is for **your notes, thoughts, or any questions** for you to discuss with your doctor.

1. Will it affect how long I live?

Lumpectomy with radiation	Mastectomy

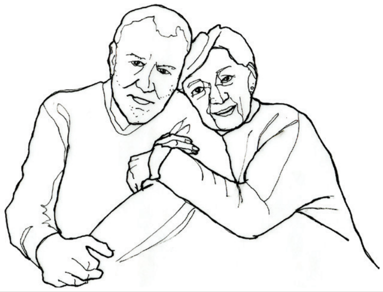

No, how long you live is the same for both surgeries.

2. Will cancer come back in the breast?

Lumpectomy with radiation	Mastectomy

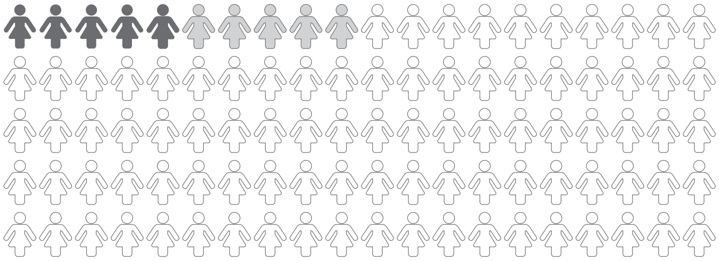

Within 10 years, breast cancer returns for about **5-10 in 100 women (5-10%)**.
This depends on the cancer stage and tumor characteristics, rather than on the type of surgery.
Please discuss your individual risks with your doctor.

3. What is removed in the breast?

Lumpectomy with radiation	Mastectomy
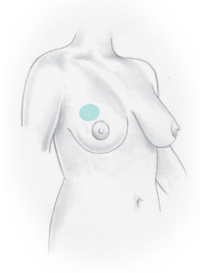	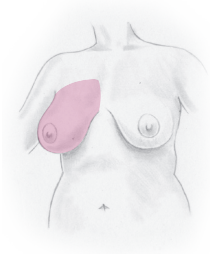
Only the cancer lump will be removed.	The whole breast will be removed.

picture option grid

4. Will I need more than one surgery?

Lumpectomy with radiation	Mastectomy
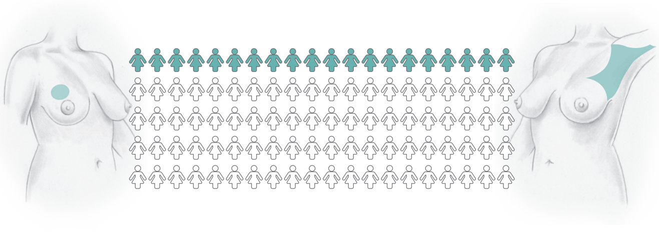	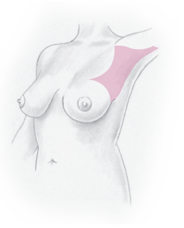 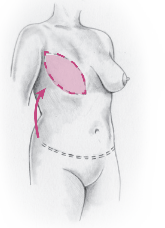
Possibly, **20 in 100 women (20%)** might need additional surgery to remove more breast tissue or lymph nodes that have cancer.	**Possibly**, if your lymph nodes have cancer. **Yes**, if you choose breast reconstruction.

5. How long will it take me to recover?

Lumpectomy with radiation	Mastectomy
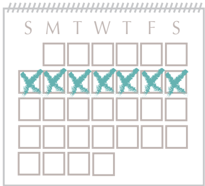	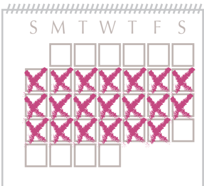

Most women are home on the same day as surgery

Lumpectomy with radiation	Mastectomy
...**but this may vary**. It will take about a week, or more, before you can resume usual activities.	...**or longer with reconstruction**. It will take several weeks, or longer, before you can resume usual activities.

6. Will I need radiation in the breast?

Lumpectomy with radiation	Mastectomy
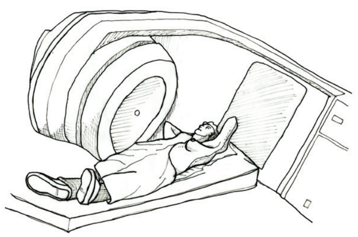 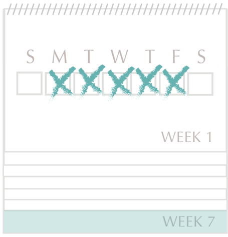	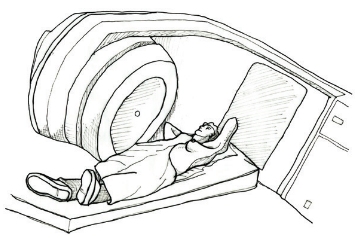 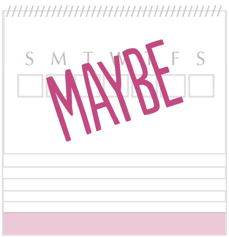
Yes, it will mean visits to the hospital **five days a week**, for up to **seven weeks** after surgery.	Radiation is **not usually given** after a mastectomy but may **sometimes** be recommended.

picture option grid

7. Will my lymph nodes be removed?

Lumpectomy with radiation	Mastectomy

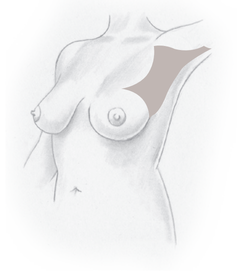

Possibly, if cancer spreads to the lymph nodes under your arm. Your doctor will discuss with you whether you should consider further treatment such as surgery or radiotherapy.

8. Will I need chemotherapy and lose my hair?

Lumpectomy with radiation	Mastectomy

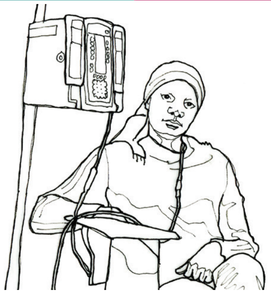

You may be offered chemotherapy, but this does not depend on the surgery you choose.
Hair loss is common after chemotherapy.

9. How much will it cost?

Lumpectomy with radiation	Mastectomy

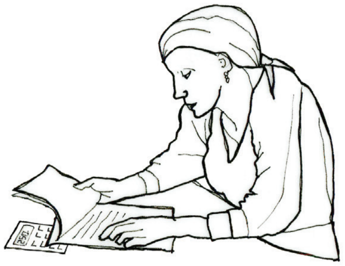

Both options have similar costs.
It is best to know what is covered by your insurance and what your out of pocket costs may be.
Don't hesitate to ask your care team about this. They will know who can answer your questions.

Notes and questions

Now that you have looked at the Picture Option Grid, this page is for your notes, thoughts or any questions for you to discuss with your doctor.

1. Will it affect how long I live?

2. Will cancer come back in the breast?

3. What is removed in the breast?

4. Will I need more than one surgery?

5. How long will it take me to recover?

6. Will I need radiation in the breast?

7. Will my lymph nodes be removed?

8. Will I need chemotherapy and lose my hair?

9. How much will it cost?

Other questions/thoughts:

Early stage breast cancer: What's right for me?
Editors: Marie-Anne Durand, Lisa Caldon, Kari Rosenkranz, Dale Collins Vidal, Stephanie Sivell, Malcolm Reed, Glyn Elwyn
Evidence document: http://optiongrid.org/cpresources/grid/evidences/9.pdf?x=Z4wDlBhPB
Publication date: 23 August 2017 Expiry date: 23 August 2019
ISBN: 978-0-9550975-6-0 License: CC BY-NC_ND 4.0 (International)
